# Apical periodontitis in inflammatory bowel disease: a meta-analysis at patient and tooth level

**DOI:** 10.3389/fdmed.2025.1553914

**Published:** 2025-02-10

**Authors:** Giusy Rita Maria La Rosa, Alejandro Ismael Lorenzo-Pouso, Vito Carlo Alberto Caponio, Mariangela Valentina Puci

**Affiliations:** ^1^ Department of General Surgery and Medical-Surgical Specialties, University of Catania, Catania, Italy; ^2^ Oral Medicine, Oral Surgery and Implantology Unit (MedOralRes Group), Faculty of Medicine and Dentistry, University of Santiago de Compostela, A Coruña, Spain; ^3^ Department of Clinical and Experimental Medicine, University of Foggia, Foggia, Italy; ^4^ Biostatistics and Clinical Epidemiology Unit, Department of Public Health, Experimental Medicine and Forensic Science, University of Pavia, Pavia, Italy; ^5^ Clinical Epidemiology and Medical Statistics Unit, Department of Medicine, Surgery and Pharmacy, University of Sassari, Sassari, Italy

**Keywords:** apical periodontitis, inflammatory bowel disease, Crohn's disease, systematic review, meta-analysis

## Abstract

Apical periodontitis (AP) is the local inflammation of periapical tissues originating from the dental pulp disease. Cumulative evidence suggests a link between oral and gastro-intestinal systems in both health and disease. In this context, the relationship between AP and inflammatory bowel diseases (IBDs) has not yet been elucidated. The aims of this systematic review and meta-analysis were to describe the prevalence of AP in patients with IBDs and evaluate the potential association between AP and IBDs. Electronic (Embase, PubMed, Scopus, Web of Science) and manual literature searches were conducted from inception to 31 October, 2023 (updated in August, 2024). Strict inclusion criteria were applied to identify observational and experimental clinical studies on AP in IBDs patients. The bias risk was assessed using the Joanna Briggs Institute critical appraisal tools and a biases' report selected from the Oxford Centre for Evidence Based Medicine Catalogue of Bias. A meta-analysis was performed to determine the pooled prevalence and risk of AP at individual and tooth level and the quality of evidence was assessed by the Grading of Recommendations, Assessment, Development and Evaluations (GRADE) approach. The search strategy identified 82 articles with 5 studies included (657 subjects, 7,142 teeth). The overall proportion of AP was 58% at patient level (95% CI = 37%–78%, *I*^2^ = 95.3%) and 7% at tooth level (95% CI = 2%–15%; *I*^2^ = 99.2%). AP was prevalent in IBDs subjects than in healthy controls, both at patient and tooth level. The pooled OR was 1.57 (95% CI = 1.04–2.35; *P* = 0.038; *I*^2^ = 20%) at patient level, and 1.91 (95% CI = 1.16–3.15; *P* = 0.011; *I*^2^ = 82%) at tooth level. A potential association between AP and IBDs is plausible, although the quality evidence was low to very low. Longitudinal and experimental studies should be conducted to better understand the relationship between these two conditions and explore any potential causative factors.

**Systematic Review Registration:**

https://www.crd.york.ac.uk/prospero/display_record.php?RecordID=411038, PROSPERO (CRD42023411038).

## 1 Introduction

Inflammatory bowel diseases (IBDs), including Crohn's disease (CD) and ulcerative colitis (UC), refer to chronic and multifactorial disorders of the intestinal mucosa characterized by an abnormal and dysregulated immune response to luminal agents in a genetically susceptible host (1, 2). The pathogenesis has been linked with a combination of genetic and environmental factors which induce an altered immune response to the gut microbiota (3, 4). UC is classified as a T_H_2 type immune disease with an upregulation of interleukin (IL)-5 while CD is considered as T_H_1 type characterized by high levels of interferon gamma (IFN-γ), IL-12, and tumor necrosis factor alpha (TNF-α) (5). Any section of gastrointestinal tract can be affected, even if the terminal ileum and the distal colon represent the most commons localization for CD and UC, respectively (6). Pharmacological treatment of IBDs includes immunosuppressive therapy such as corticosteroids (7) and biological medications, indicated for patients not responding to conventional treatment (8). Clinical manifestations are abdominal pain, weight loss, rectal bleeding and asthenia. Yet, extraintestinal manifestations are frequent and regard renal, musculoskeletal, dermatologic, pulmonary and oral manifestations (9, 10). Oral manifestations occur in 4%-16% of patients with IBD with deep ulcerations, angular cheilitis, glossitis (11, 12), caries (13) and periodontitis (13, 14).

Apical periodontitis (AP) is a chronic inflammatory disease of the periradicular tissues generated by the progression of microbial infection from the dental pulp to the periodontium. The chronic inflammation promotes the activation of host immune defense with the consequent persistent tissue damage of periradicular structures (15, 16). In addition to being a local infection, many microbes and toxins of AP may enter the bloodstream through the root canal system (17, 18). In addition, AP is able to modulate the systemic immune response by modifying the levels of inflammatory cytokines (19, 20). In recent years, the relationship between endodontic status and systematic disease has been widely investigated, suggesting an association between AP and several inflammatory systemic diseases including diabetes mellitus (21, 22) and coronary heart disease (23, 24). However, as concerns for IBD, few studies have been conducted and no clear scientific evidence has been provided on the relationship between AP and IBDs (25–29).

The aim of this systematic review and meta-analysis is to provide a comprehensive overview of the prevalence of AP among patients diagnosed with IBDs. Additionally, the study aimed to assess the potential association between these two conditions.

## 2 Methods

The present review was reported following the Preferred Reported Items for Systematic Reviews and Meta-analyses (PRISMA) guidelines (30) and the protocol was registered *a priori* in PROSPERO (CRD42023411038).

The research strategy was based on the PICOS (Population, Intervention/Exposure, Comparator, Outcome, Study design) approach as follows:

Population (P) — patients (18–80 years) with IBDs, including CD and UC, diagnosed based on clinical criteria, endoscopy, histopathological examination, or other established diagnostic methods; dentate human subjects with primary and permanent dentition;
Exposure (E) —inflammatory bowel disease;
Comparison (C) — healthy subjects without IBDs;
Outcome (O) — prevalence of AP (diagnosed radiographically using periapical radiographs, panoramic radiographs, or computerized tomography) in patients with IBDs, at patient and tooth level. Association between AP and IBDs measured as odds ratio (OR), at patient and tooth level.
Study design (S) — Experimental (i.e., randomized controlled studies) and observational (i.e., cross-sectional, case-control and cohort design).

### 2.1 Search strategy

Four electronic databases (Embase, PubMed, Scopus, and Web of Science) were used to identify articles published until 31 October, 2023. An update was performed on Aug 2024. The following keywords, combined in different strings, were used to search articles: “root filled tooth”, “periradicular lesion”, pulpitis, “dental pulp disease”, “root canal therapy”, “periapical periodontitis”, “apical periodontitis”, “inflammatory bowel disease”, “crohn's disease”, “crohn disease”, “ulcerative colitis”, “colitis, ulcerative”. The specific search strategy applied for each database is reported in Supplementary Table S1.

Pertinent reviews published from January 2015 to October 2023 were screened for additional studies. Citation chasing of the included studies was performed in Google Scholar. A manual search was also conducted through the most relevant journals: *Journal of Endodontics, International Endodontic Journal, Clinical Oral Investigations, Odontology, Australian Endodontic Journal, BMC Oral Health*. A grey literature search was conducted on the American Association of Endodontists and the American Dental Association websites. No date and language restrictions were applied.

### 2.2 Inclusion criteria

(1) Experimental (i.e., randomized controlled studies) and observational (i.e., cross-sectional, case-control and cohort design) studies; (2) studies that were conducted among adults (18–80 years old) with IBDs (as diagnosed using any recognized diagnostic criteria); (3) information on the number of teeth with AP and number of root filled teeth (RFT) with AP on patient level or tooth level or both; (4) studies with control group on healthy subjects.

### 2.3 Exclusion criteria

(1) Studies without data on prevalence estimates of AP; (2) studies without radiographic assessment; (3) article type (i.e., editorials, commentaries, letters, conference abstracts, preprint articles, and unpublished data); (4) study design (i.e., case report, case series, *in-vitro*, *ex-vivo*, and animal studies).

### 2.4 Study selection

A first assessment of record titles and abstracts was carried out by two investigators independently and supervised by a third, following the inclusion and exclusion criteria. Afterwards, studies satisfying the inclusion criteria were further screened via full-text.

The screening process was conducted by the two same independent reviewers blinded to each other's' decisions, and any disagreement was resolved by discussion and if necessary, by the decision of a third investigator. All duplicates were removed. Studies were managed by using EndNote program (EndNote X9; Thomson Reuters, New York, NY).

### 2.5 Data extraction

The data extraction process was performed by two independent reviewers with a standard data extraction form adopted from the Joanna Briggs Institute (JBI) model (31). The items included bibliographic notes, population details, outcomes, statistical analysis, main findings, limitations and conclusions of the study.

### 2.6 Quality assessment

The quality of the included studies was performed by two independent reviewers using JBI quality assessment tools (Supplementary Table S2) and a report of biases selected from the Oxford Centre for Evidence Based Medicine *Catalogue of Bias* [i.e., Reporting, Spin, Selection, Information, All's well literature, Confirmation, Confounding, Hot stuff, Prevalence- incidence (Neyman), Substitution game, Volunteer, Wrong sample size] (32, 33). Any disagreement was resolved by discussion; no intervention of a third examiner was necessary. When evaluating studies using the JBI critical appraisal tool, studies were judged as having a “low risk of bias” if all items scored “yes”; “some concerns of bias” if at least one scored “unclear” and “high risk” if at least one scored “no”. Based on JBI score and biases report, studies were classified as at low risk of bias, some concerns and high risk (34). No studies were excluded on the basis of quality assessment or risk of bias findings. Moreover, deviations from protocol and discrepancies in the data reporting were also assessed (34–36).

### 2.7 Concordance of protocol and full-text publication

The age limit for inclusion was extended from 70–80 years to ensure that the widest possible audience was included. Subgroup analyses (by gender and type of disease) were initially intended as part of the protocol registration. Nevertheless, these subgroup analyses could not be carried out due to the insufficient number of studies identified in the review.

### 2.8 Data synthesis and statistical analysis

Descriptive statistics were used to summarize main findings and demographic characteristics of included studies. The data analysis on prevalence of AP in IBDs patients and the estimated risk was performed both on patient level and tooth level, when available. The bibliographic and demographic characteristics of the included studies were tabulated for IBDs and control groups. The findings were also narratively synthetized and described.

Meta-analytic estimates were computed and described with pooled and heterogeneity indicators. Forest plots were used to represent study variability with 95% confidence intervals (CI) for prevalence of AP and the risk AP-IBDs.

To assess the heterogeneity among studies the inconsistency indicator (*I*^2^) was calculated, where an *I*^2^ value > 50% indicated substantial heterogeneity. Thus, fixed- and random-effects models were computed keeping into consideration the expected between-study heterogeneity. Given that the review involved fewer than 10 studies, the potential publication bias was not explored (35, 37). A two-tailed *p*-value less than 0.05 was considered statistically significant. Data analyses were performed using STATA17 (StatsCorp, College Station, Texas, USA) and R (version 4.3.1) software.

### 2.9 Grading of recommendations assessment, development and evaluation

The certainty of the body of evidence for the outcome of association between IBDs and AP at patient and tooth level was determined by using Grading of Recommendations Assessment, Development and Evaluation (GRADE) (38).

## 3 Results

### 3.1 Literature search

**Figure 1:**
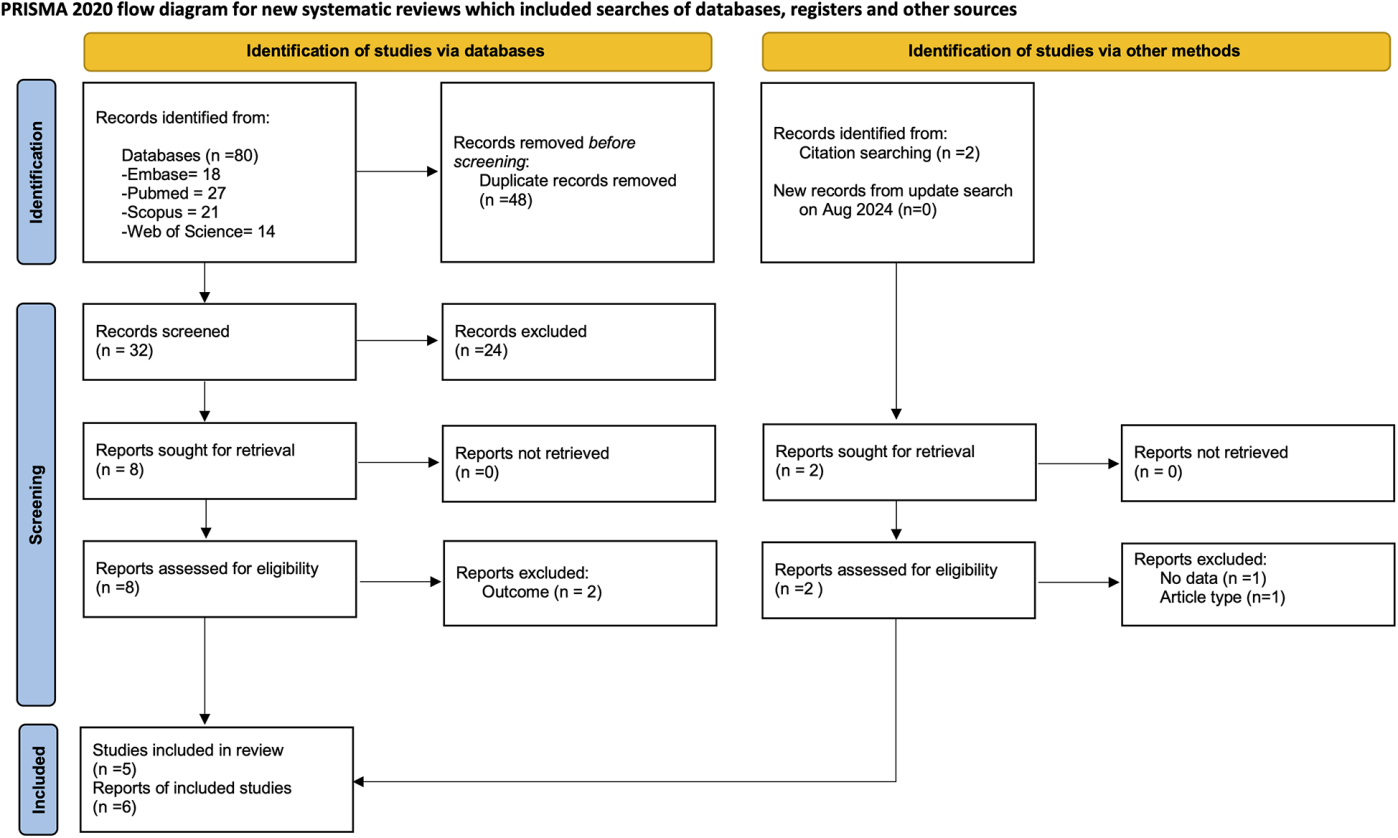
Flow diagram for search process according to the preferred reported items for systematic reviews and meta-analyses (PRISMA).

Electronic database and manual search retrieved 82 records from different sources, 34 potential studies were identified for screening. After examining these articles, 10 were suitable for full text and 4 were excluded. A total of 5 studies with 6 publications met the inclusion criteria (25, 27–29, 39, 40) with a total of 657 subjects and 7,142 teeth (Figure 1). No new studies were retrieved in the updated search.

The articles excluded after full-text assessment and the reason for their exclusion are listed in Supplementary Table S3. Two studies were published using the same data (27, 28), but only one (27) was included in the present study. Concerning patient-level a total of four studies were included (25, 27, 29, 40). For tooth-level analysis, two studies were included (39, 40).

### 3.2 Characteristics of the included studies

The selected five studies were published between 2017 and 2023 (25, 27, 29, 39, 40) (Table 1) The study design was clearly reported in each study: three were case-control studies (27, 29, 40), while two were retrospective studies based on medical records (25, 39). Studies were conducted in 3 countries worldwide: 2 in Italy (25, 39), 2 in Spain (27, 29), one in United Kingdom (40). The sample size in the IBDs groups ranged from 16–110 at patient-level and from 400–1,758 at tooth-level (Table 2). All studies were monocentric.

**Table 1:** Summary of included studies.

Author & year	Title	Study design	Setting	Study period	Disease years at dental evaluation	Study aim	Assessment method
Allihaibi et al. (40)	Prevalence of apical periodontitis in patients with autoimmune diseases: A case-control study	Case-control	Dental clinics at Guy’s and St Thomas’ NHS Foundation Trust (London, UK)	January 2012–December 2020	NR	To compare the prevalence of AP in patients affected by autoimmune disorders including IBD.	Dental panoramic tomograms
Ideo et al. (39)	Prevalence of Apical Periodontitis in Patients with Autoimmune Diseases under Immunomodulators: A Retrospective Cohort Study	Retrospective cohort	University Dental Clinic (Cagliari, Italy)	January 2017–December 2020	NR^a^	To investigate the prevalence of AP in patients affected by autoimmune diseases taking biologic medications BMs.	Panoramic radiograph
Piras et al. (25)	Prevalence of Apical Periodontitis in Patients with Inflammatory Bowel Diseases: A Retrospective Clinical Study	Retrospective cohort	University Dental Clinic (Cagliari, Italy)	June 2012–July 2015	12 ± 7.5	To assess the prevalence of AP and the oral health status in patients with IBD treated with immunomodulators, particularly BMs.	Periapical radiographs
Poyato-Borrego et al. (27)	High Prevalence of Apical Periodontitis in Patients with Inflammatory Bowel Disease: An Age- and Gender- matched Case-control Study	Case-control	San Juan de Dios Hospital (Seville, Spain)	2017–2018	NR	To determine the frequency of AP and RCT in IBD patients and healthy control subjects.	Digital orthopantomography
Segura-Sampedro et al. (29)	Periapical and endodontic status of patients with inflammatory bowel disease: Age- and sex-matched case–control study	Case-control	Hospital Odontòlogic de Bellvitge, Universitat de Barcelona (Barcelona, Spain)	2018–2021	CD = 13.7 ± 8.3	To evaluate the possible association between IBD and the prevalence of AP and RCT.	Digital panoramic radiographs
					UC = 14.7 ± 10.0		

AP, apical periodontitis; BM, biologic medications; CD, Crohn's disease; IBDs, inflammatory bowel diseases; NR, not reported; RCT, root canal treatment; UC, ulcerative colitis.

^a^ Data aggregated for all included autoimmune diseases; not available specifically for IBD.

**Table 2:** Demographic characteristics and study outcomes of included studies.

Author & year	Sample size (patients)	Sample size (teeth)	Age (years, mean ± SD)	Sex, *n* (%)	IBDs treatment	Type of disease *n* (%)	AP *n* (%) (patients)	RFT with AP *n* (%) (patients)	AP *n* (%) (teeth)	RFT with AP *n* (%) (teeth)
Allihaibi et al. (40)	Control: 89	Control: 2,329	Control: 49.5 ± 14.7	Control: Female 52 (58.4)	NR^a^	NR	Control: 66 (74.2)	NR	Control: 244 (10.5)	NR
							IBDs: 12 (75)		IBDs: 59 (14.8)	
				Male 37 (41.6)						
	IBDs: 16	IBDs: 400	IBDs: NR	IBDs:NR						
Ideo et al. (39)	Control: 99	Control: 2,655	Control: 48.0 ± 15.8	Control: Female 57 (57.6)	NR^a^	NR	Control: 46 (46.5)	NR	Control: 72 (2.7)	NR
	IBDs: 69	IBDs: 1,758								
			IBDs:NR				IBDs: NR		IBDs: 113 (6.4)	
				Male 42 (42.4)						
				IBDs:NR						
Piras et al. (25)	Control: 110	NR	Control: 41 ± 13.1	Control: Female 57 (52)	BMs = 74	NR	Control: 65 (59)	NR	NR	NR
	IBDs: 110				Corticosteroids = 36					
			IBDs: 46 ± 13.8				Female 56 (51)			
				Male 53 (48)						
							Male 75 (68)			
				IBDs: Female 49 (44.6)			IBDs: 70 (64)			
							Female 28 (59)			
				Male 61 (55.4)						
							Male 42 (69)			
Poyato-Borrego et al. (27)	Control: 54	NR	Control: 41 ± 13.8	Control: Female 23 (42.6)	Corticosteroids = 6	CD 28 (51.8)	Control: 9 (16.7)	Control: 8 (36.4)	NR	NR
	IBDs: 54				BMs = 5					
			IBDs: 43.1 ± 14.0		5-aminosalicylic acid = 44	UC 26 (48.1)	IBDs: 19 (35.2)	IBDs: 14 (48.3)		
				Male 31 (57.4)						
					Immuno-modulators = 24					
				IBDs: Female 23 (42.6)						
				Male 31 (57.4)						
Segura-Sampedro et al. (29)	Control: 28	NR	Control: 58.6 ± 11.9	Control: Female 20 (71.4)	NR	CD 13 (46.4)	Control: 17 (60.7)	Control: 3 (10.7)	NR	NR
	IBDs: 28									
			IBDs: 59.1 ± 10.9			UC 15 (53.6)	IBDs: 23 (82.1)	IBDs: 15 (53.6)		
				Male 8 (28.6)						
				IBDs: Female 20 (71.4)						
				Male 8 (28.6)						

AP, apical periodontitis; BMs, biological medications; CD, Crohn's disease; CI, Confidence interval; IBDs, Inflammatory bowel diseases; NR, Not reported; RFT, root filled teeth; SD, Standard deviation; UC, ulcerative colitis.

^a^ Data aggregated for all included autoimmune diseases; not available specifically for IBD.

### 3.3 Synthesis of the results

The IBDs patients were predominantly men in two studies (25, 27) and females in one study (29) (Table 2). The mean age of IBDs patients was 49.4 ± 8.52 years, and was reported only in three studies (25, 27, 29).

Periodontal status was inconsistently assessed across the included studies. In Piras et al. (2017) (25), probing depth was measured, but the data were not reported. Two studies, Allihaibi et al. (40) and Poyato-Borrego et al. (27), did not evaluate periodontal status. Segura-Sampedro et al. (29) assessed periodontal disease radiographically, defining periodontitis as alveolar bone loss ≥4 mm, with most participants being free of the condition (46/56). In Ideo et al. (39), patients with periodontitis were excluded, and while probing depth was recorded, these data were not reported.

The DMFT score was reported in two studies (25, 39), and in both, no significant differences emerged between the control group and patients with IBDs. A third study (40) reported a higher DMFT score for the group with autoimmune diseases, including IBDs. The mean DMFT score in patients affected by IBD was 16.5, compared to 14.0 ± 8.6 in the control group.

In general, patients with IBDs were recruited without additional systemic conditions such as diabetes, metabolic syndrome, or cardiovascular disease in two studies (25, 29). Two studies included patients diagnosed with autoimmune diseases, such as IBD, rheumatoid arthritis, and psoriasis (39, 40). For the control group, the same criteria were applied, except for the diagnosis of IBD (i.e., healthy subjects without IBD).

Three studies (25, 27, 29) included patients with both CD and UC. In one of these studies, no significant differences were observed among patients with UC and those with CD in the number of teeth with AP, the number of RFT, or the number of RFT with AP (*P* > 0.05) (27). However, the other two studies did not provide stratified data for the two diseases.

The remaining two studies (39, 40) included patients with autoimmune diseases, which also covered IBDs, but did not provide further details regarding the specific type of IBD.

All included studies reported higher prevalence of AP in IBDs patients compared to the healthy subjects with values ranging from 21.4% (29)–75% (39). Evidence of AP was performed by digital panoramic radiographs in almost all studies (27, 29, 39, 40); one by periapical radiographs (25). Periapical index (PAI) was determined in four studies (25, 27, 29, 39).

The association of AP and IBDs compared to the healthy controls resulted significantly higher at patient-level in one study (27), not significant in three studies (25, 29, 40), not reported in one study (39). A gender-analysis was conducted only in one study (25) which reported a significantly higher number of teeth with AP in women with IBDs compared to healthy women; yet, the association AP-IBDs was not statistically significant.

The percentage of RFT and RFT with AP at patient-level was reported in two studies (27, 29) and not specified in three studies (25, 39, 40). No significant differences were detected in the number of subjects with 1 or more RFT neither in RFT with radiological signs of AP between the IBDs and control groups in Poyato-Borrego et al. (27) while a significant higher number of patients with RFT and RFT-AP was detected in IBDs patients in the study of Segura-Sampedro et al. (29).

The quality of root canal treatment and coronal restoration of endodontically treated teeth was reported in two studies (25, 39) and not specified in the remaining (27, 29, 40). Of note, the quality of coronal and endodontically restoration was judged adequate in about 60% of both IBDs and control cases in one study (25) whereas was considered adequate only in about 10% of cases in the study of Ideo et al. (39).

### 3.4 Meta-analysis findings

#### 3.4.1 AP and RFT in patient level

**Figure 2:**
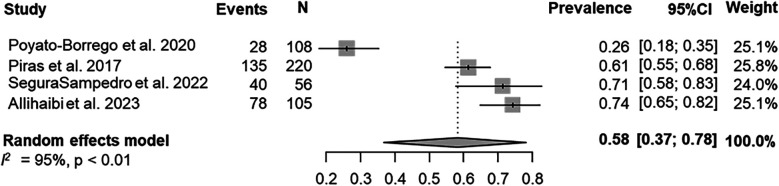
Overall prevalence of AP in the whole population (IBDs and healthy patients)—patient level.

**Figure 3:**
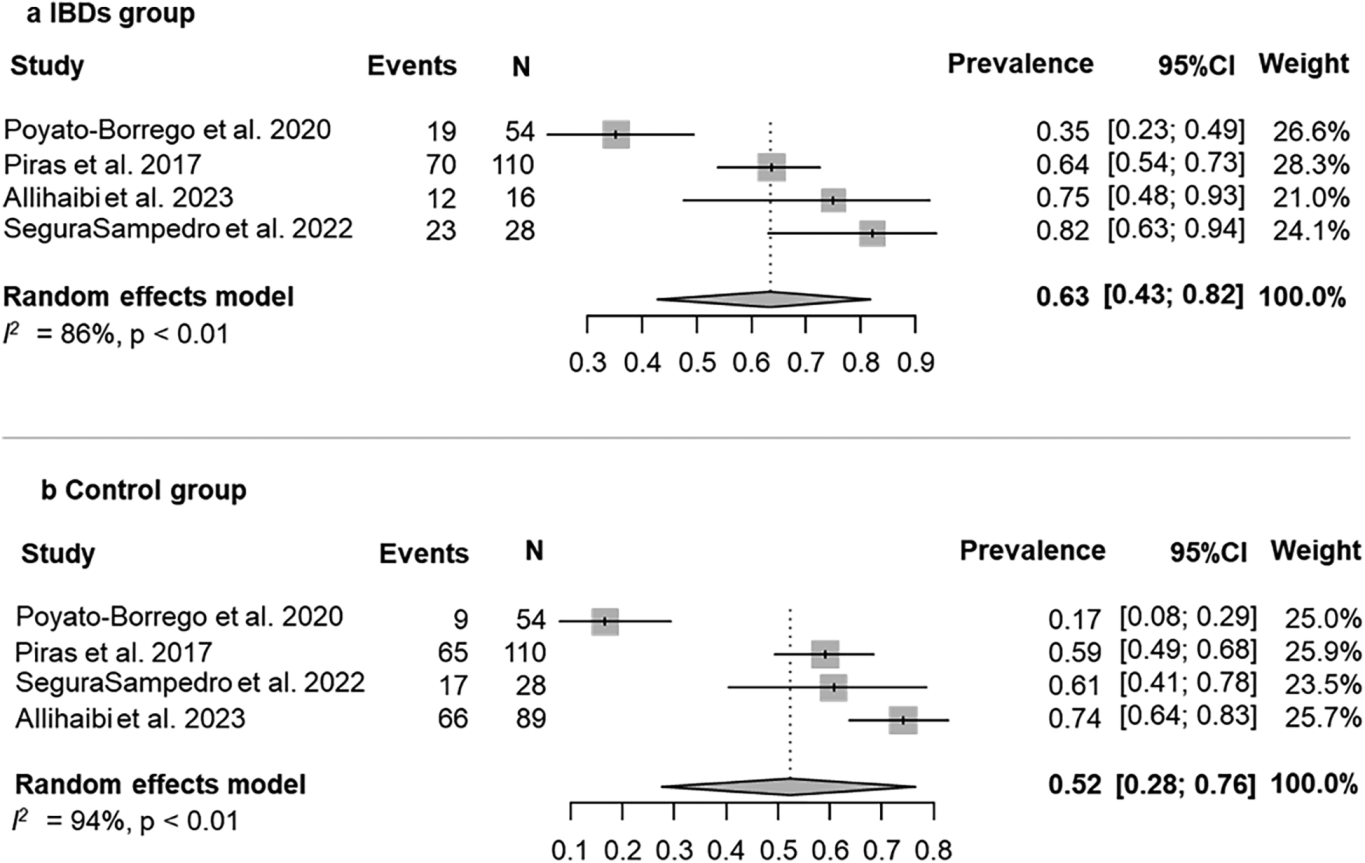
Prevalence of AP in the IBDs **(a)** and control group/healthy subjects **(b)**-patient level.

**Figure 4:**
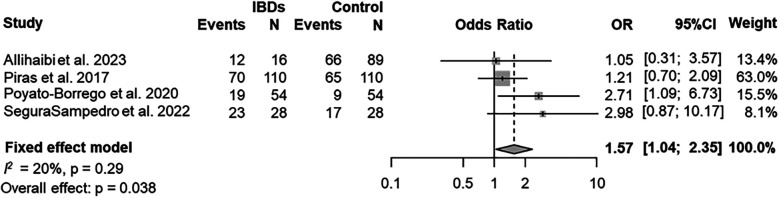
Pooled OR for AP in IBDs/healthy subjects—patient level.

The prevalence of AP was evaluated in 4 (80%) studies (25, 27, 29, 40). According to the results of the pooled data for both IBDs and healthy patients, the overall prevalence of patients with AP was 58% (95% CI = 37%–78%; Figure 2). The prevalence of AP was higher among patients with IBDs than those from control group (63%; 95% CI = 43%–82%; VS. 52%; 95% CI = 28%–76% respectively; Figures 3a,b). The AP was significantly associated with IBDs, with a pooled OR of 1.57 (95% CI = 1.04–2.35; *P* = 0.038; Figure 4).

Concerning RFT, it was evaluated by two studies (27, 29) with an overall pooled prevalence of 46% (95% CI = 35%–57%; Supplementary Figure S1). RFT was prevalent in IDBs group (57%; 95% CI = 43%–71%) compared to control group (30%; 95% CI = 16%–47%; Supplementary Figures S2a,b). However, no significant association was found for RFT-AP and IDBs: pooled OR was 3.26 (95% CI = 0.71–15.0; *P* = 0.13; Supplementary Figure S3).

#### 3.4.2 AP in tooth level

The pooled proportion of AP was evaluated by 2 out of 5 (40%) studies (39, 40). The overall prevalence of patients with AP was 7% (95% CI = 2%–15%; Supplementary Figure S4). Similarly, to patient level analysis, AP was prevalent in IBDs patients than in control group (10%; 95% CI = 3%–20%; VS. 6%; 95% CI = 1%–16% respectively; Supplementary Figures S5a,b). Significant association was found for AP and IBDs, with a pooled OR of 1.91 (95% CI = 1.16–3.15; *P* = 0.011; Supplementary Figure S6).

### 3.5 Quality assessment

Bias assessment for each study is summarized in Table 3. The complete JBI critical appraisal for each study is shown in Supplementary Figure S7. Overall, two studies were judged with some concerns (25, 39) and three at high risk of bias (27, 29, 40).

**Table 3:** Overall risk of bias assessment following joanna briggs institute critical (JBI) appraisal checklist and catalogue's bias list.

	Included studies				
	Allihaibi et al. (40)	Ideo et al. (39)	Piras et al. (25)	Poyato-Borrego et al. (27)	Segura-Sampedro et al. (29)
Catalogue’s bias					
Reporting	No protocol	No protocol	No protocol	No protocol	No protocol
			Text discrepancies		
Spin	No null hypothesis	Emphasis on not significant results	Emphasis on not significant results	None	None
		No null hypothesis	No null hypothesis		
Selection	No randomization (case-control)	No randomization (medical records)	No randomization (medical records)	No randomization (case-control)	No randomization (case-control)
Information	None	No blinding examiner reported	No blinding examiner reported	None	None
All’s well literature	None	None	None	None	None
Confirmation	None	None	None	None	None
Confounding	Quality of coronal restorations/root filling not evaluated	None	None	Quality of coronal restorations/root filling not evaluated	Quality of coronal restorations/root filling not evaluated
Hot stuff	None	Emphasis on not significant results	Emphasis on not significant results	None	None
Prevalence- incidence (Neyman)	Case-control study	Retrospective study	Retrospective study	Case-control study	Case-control study
Substitution game	None	None	None	None	None
Volunteer	None	None	None	Volunteers recruited	Volunteers recruited
Wrong sample size	None	None	No sample size calculation		None
JBI assessment					
	High	Low	Low	High	High
OVERALL					
	High	Some concerns	Some concerns	High	High

### 3.6 GRADE evaluation

The quality of evidence assessed by the GRADE approach ranged from low to very low for the association IBDs-AP at patient and tooth level respectively (Supplementary Table S4).

## 4 Discussion

### 4.1 Summary of evidence

IBDs association with different oral health diseases has been previously described (41–43). The current findings showed that patients with IBDs had significantly increased odds of suffering AP than patients without IBDs at the patient level. One interesting finding is that the association between AP proportion and IBDs was significant also at the tooth level despite the potential diluting effect due to individual variation. On the other hand, the pooled analysis did not reveal a significant association between AP in RFT and IBDs. In RFT, periapical lesions should be interpreted carefully because they may represent either a healing lesion or an apical scar (44). It has been hypothesized that healing process of the periapical osteolytic lesion may be delayed due to alterations in the OPG/RANKL/RANK system (45) and enhanced activation of NLRP3 inflammasome (46) in patients with IBD. Thus, it is pivotal for clinical studies to report the time elapsed between the RFT and the diagnosis of AP.

The current results are consistent with previous systematic reviews that have attempted to estimate the prevalence of AP in the general population (47, 48). In addition, the present findings closely align with the subgroup analysis estimates of AP prevalence at the patient and tooth levels when stratifying the group with systemic conditions (47). This finding supports the hypothesis that there is a potential relationship between AP and IBDs, and it adds to the existing literature exploring the connection between oral health and systemic diseases.

The association between IBDs and periodontitis demonstrated a bidirectional relationship (49, 50). It is plausible hypothesizing a similar mechanism also for AP. Individuals with IBDs may face a heightened risk of developing AP due to the inflammatory response and immune system dysregulation associated with IBDs (25).

Apical periodontitis triggers TH1 response causing bone destruction (51) and TH2 response aiding post-root canal treatment repair (52). Genotype may link both conditions (27). AP might heighten IBD risk via chronic oral infections inducing systemic inflammation (53), implicating oral-gut interaction through microbiome and immune cell pathways (54). Resemblance between dysbiotic gut microbiome in IBD and oral microbiome, termed ‘oralization’ (55, 56), suggests potential mechanisms like bacterial transmission, immune cell migration, and genetic factors (57), yet necessitates further investigation for clarity on relationship direction.

The different radiographic examinations vary in their sensitivity and specificity for detecting periapical lesions (58, 59). Studies that rely solely on panoramic radiographs may underestimate the prevalence of AP lesions, as this method has lower sensitivity but higher specificity (60). Yet, the distinction between periapical and panoramic radiographs in detecting periapical lesions was reported to be not statistically significant (58). On the other hand, panoramic radiography offers several advantages over periapical radiographs. It allows for the visualization of all teeth in a single comprehensive image, thereby reducing the patient's exposure to radiation (61). Additionally, the delayed appearance of inflammatory processes on radiographic examination and the reliability of radiographic interpretation can further contribute to variability in the findings (62). Two studies (25, 39) did not report blinding of examiners potentially introducing information bias.

The presence or absence of pulpal pathologies associated with AP lesions was not specifically reported in the included studies. However, as the diagnosis of AP was performed using radiographs, it is reasonable to assume that the affected teeth were predominantly asymptomatic necrotic teeth or previously endodontically treated teeth. This assumption aligns with the data reported in two of the four studies included in the meta-analysis (27, 29). It is important to note that periapical lesions of non-odontogenic origin are extremely rare and are typically part of broader systemic conditions. While these lesions are unlikely to have significantly influenced our findings, their presence cannot be entirely ruled out. Future studies should aim to collect detailed data on pulpal pathologies and differentiate odontogenic from non-odontogenic lesions to better understand their potential impact on the observed associations.

Many IBD treatments, like corticosteroids and biologics, target the immune system to reduce inflammation (63). Both conditions involve similar immune pathways and pro-inflammatory cytokines (53), suggesting these drugs might impact dental infections (64). Wang et al. found that cell membrane vesicles enriched with CXCR4 can target both ulcerative colitis and AP (65). While IBD treatment could potentially confound this relationship, it might dilute the true association. Any bias introduced likely leans towards null value.

As some confounders have not been excluded, the results should be carefully evaluated. A number of relevant factors influencing periapical status and root canal treatment [RCT] prevalence have not been addressed, such as educational level, socioeconomic status, trauma history and quality of coronal restoration and root filling. The quality of root canal fillings and coronal restorations have been associated with the prevalence of chronic AP (66). However, these factors were not considered in assessing radiolucent periapical lesions in most of the included studies (27, 29, 40). The lack of consideration for confounding factors may explain the variability in retrieved studies. The management of confounding factors can be challenging, especially when their impacts on AP are likely to be multifactorial.

Moreover, there is a risk of introducing selection bias as studies might inadvertently include more individuals with RFT-AP in the control group, given their higher likelihood of receiving root canal treatments for dental issues, potentially leading to an understated association between IBD and AP.

### 4.2 Comparison with previous reviews. What’s new?

A previous systematic review assessed the association between pulpal-periapical pathology and autoimmune diseases, including IBDs (67). They reported that an association between AP and autoimmune diseases may be plausible, although most studies showed non-significant associations. A recent systematic review has also reported similar results regarding the potential association between apical periodontitis and gastrointestinal disorders, such as inflammatory bowel disorders (68). The mentioned reviews differed from our systematic review in the inclusion criteria, outcomes (lack of prevalence data), and absence of meta-analysis and GRADE assessment. Moreover, we performed an additional analysis specifically at the level of root-filled teeth.

Another systematic review with meta-analysis on the same topic has also been conducted (69). This review, however, also differs in several key aspects. First, prevalence data were not among the outcomes analyzed in the other review. Second, our study utilized a comprehensive bias assessment tool, which accounts for the main biases often overlooked in the tools used in other bias evaluations. Additional differences include the fact that we conducted a meta-analysis both at the tooth and patient levels to evaluate the potential diluting effect due to individual variation.

### 4.3 Limitations and strengths

The limited number of eligible studies may have affected statistical power and result reliability (70), cautioning interpretation of pooled estimates and urging further investigation. Retrospective case-control designs introduce selection biases (71), hindering cause-effect relationship establishment (72). Concerns or high risk of biases in included studies led to lower GRADE scores, indicating low to very low-quality evidence. Heterogeneity due to the small study number was addressed through random effects estimates for cautious evaluation (73, 74).

The current knowledge has several limitations that should be addressed in future research. One significant limitation is the lack of data on participants' oral hygiene status in the included studies. Oral hygiene is a crucial determinant of oral health and may influence the prevalence of oral diseases. Future studies should aim to collect and report comprehensive information on oral hygiene to provide a clearer understanding of its potential role in the relationship between AP and systemic conditions such as IBDs.

While some studies may have reported years since diagnosis (25, 29), this information was not uniformly provided or analyzed in the meta-analysis. Collecting detailed data on the chronicity of IBDs could provide valuable insights into its potential role in the association between AP and IBDs. Additionally, there was a lack of standardization regarding the classification of IBDs, their treatment, and the types of IBD (e.g., UC vs. CD) reported in the included studies, which limits the generalizability of the findings.

Another limitation is the absence of a uniform assessment of dental histories and DMFT scores across the included studies. These parameters are critical for understanding the dental health background of participants and could influence the observed association between AP and IBDs.

Furthermore, the included studies employed different radiographic methods (e.g., panoramic radiographs, periapical radiographs), which vary in their sensitivity and specificity for detecting AP. This variability may have contributed to differences in the detection and reporting of AP lesions. Future studies should aim for greater consistency in radiographic assessments to improve comparability.

On a positive note, this meta-analysis adhered to rigorous quality standards, ensuring transparency and reproducibility, and all primary studies consistently reported an increased risk, adding strength to the observed association. Notably, this is the first systematic review with meta-analysis at both patient and tooth level providing an updated assessment of the prevalence and risk of AP in IBDs patients.

### 4.4 Clinical implications

In line with translational research in dentistry, dentists should screen IBD patients for pulpitis and periapical lesions, potentially enhancing oral health outcomes (29).

## 5 Conclusions

Within the limitations of the present systematic review and meta-analysis, AP seems to be frequent among IBD patients. Despite a low to very low quality of evidence, our findings suggest a possible association between the two pathologies which requires further investigations. Future high-quality experimental and longitudinal studies are recommended to better understand the causality relationship in the studied diseases.

## Data Availability

The raw data supporting the conclusions of this article will be made available by the authors, without undue reservation.
