# Supplementary material for: Apical periodontitis in inflammatory bowel disease: a meta-analysis at patient and tooth level
Source: Front Dent Med. 2025 Feb 10;6:1553914. doi: 10.3389/fdmed.2025.1553914 (PMC11847799; doi:10.3389/fdmed.2025.1553914)

**1. SUPPLEMENTARY MATERIALS**

**Table S1.** Search strategy adopted for each database

| **Database** | **Search strategy** |
| --- | --- |
| **Pubmed** | ((((root filled tooth) OR (periradicular lesion) OR ((pulpitis) OR (pulpitis[MeSH Terms])) OR ((dental pulp disease) OR (dental pulp disease[MeSH Terms])) OR ((root canal therapy) OR (root canal therapy [MeSH Terms]))) OR ((periapical periodontitis) OR (periapical periodontitis [MeSH Terms]))) OR ((apical periodontitis) OR (apical periodontitis[MeSH Terms]))) AND ((inflammatory bowel disease) OR (inflammatory bowel disease[MeSH Terms]) OR ((((crohn's disease[MeSH Terms]) OR (crohn's disease)) OR (crohn disease[MeSH Terms])) OR (crohn disease)) OR (crohn disease*) OR (((ulcerative colitis[MeSH Terms]) OR (ulcerative colitis)) OR (colitis, ulcerative[MeSH Terms])) OR (colitis, ulcerative)) |
| **Scopus** | (TITLE-ABS-KEY ((("root filled tooth") OR ("periradicular lesion") OR pulpitis OR ("dental pulp disease") OR ("root canal therapy") OR ("periapical periodontitis") OR ("apical periodontitis"))) AND TITLE-ABS-KEY ((("inflammatory bowel disease") OR ("crohn's disease") OR ("crohn disease") OR ("ulcerative colitis") OR ("colitis, ulcerative")))) |
| **Web of Science** | ("root filled tooth" OR "periradicular lesion" OR pulpitis OR "dental pulp disease" OR "root canal therapy" OR "periapical periodontitis" OR "apical periodontitis") (All Fields) AND ("inflammatory bowel disease" OR "crohn's disease" OR "crohn disease" OR "ulcerative colitis" OR "colitis, ulcerative") (All Fields) |
| **Embase** | ("apical periodontitis" OR "pulp disease" OR pulpitis) AND ("inflammatory bowel disease" OR "Crohn´s disease" OR "ulcerative colitis") |

**Table S2.** Joanna Briggs Institute critical (JBI) appraisal checklist for case-control and cohort studies

| **JBI QUESTIONS FOR QUALITY ASSESSMENT OF COHORT STUDIES** | **JBI QUESTIONS FOR QUALITY ASSESSMENT OF CASE-CONTROL STUDIES** |
| --- | --- |
| 1. Were the two groups similar and recruited from the same population? | 1. Were the groups comparable other than the presence of disease in cases or the absence of disease in controls? |
| 1. Were the exposures measured similarly to assign people to both exposed and unexposed groups? | 1. Were cases and controls matched appropriately? |
| 1. Was the exposure measured in a valid and reliable way? | 1. Were the same criteria used for identification of cases and controls? |
| 1. Were confounding factors identified? | 1. Was exposure measured in a standard, valid and reliable way? |
| 1. Were strategies to deal with confounding factors stated? | 1. Was exposure measured in the same way for cases and controls? |
| 1. Were the groups/participants free of the outcome at the start of the study (or at the moment of exposure)? | 1. Were confounding factors identified? |
| 1. Were the outcomes measured in a valid and reliable way? | 1. Were strategies to deal with confounding factors stated? |
| 1. Was the follow up time reported and sufficient to be long enough for outcomes to occur? | 1. Were outcomes assessed in a standard, valid and reliable way for cases and controls? |
| 1. Was follow up complete, and if not, were the reasons to loss to follow up described and explored? | 1. Was the exposure period of interest long enough to be meaningful? |
| 1. Were strategies to address incomplete follow up utilized? | 1. Was appropriate statistical analysis used? |
| 1. Was appropriate statistical analysis used? |  |

**Table S3.** List of excluded studies after full text assessment

| **Reference** | **Reason for exclusion** |
| --- | --- |
| 1. Cotti, E., Mezzena, S., Schirru, E., Ottonello, O., Mura, M., Ideo, F., Susnik, M., & Usai, P. (2018). Healing of Apical Periodontitis in Patients with Inflammatory Bowel Diseases and under Anti-tumor Necrosis Factor Alpha Therapy. Journal of endodontics, 44(12), 1777–1782. | Outcome |
| 2. Dolan, S., & Rae, E. (2023). Apical periodontitis and autoimmune diseases-should we be screening patients prior to therapy?. Evidence-based dentistry, 24(2), 64–65. | Study design |
| 3. Heikkilä, P., Niskanen, L., But, A., Sorsa, T., & Haukka, J. (2022). Oral health associated with incident diabetes but not other chronic diseases: A register-based cohort study. Frontiers in oral health, 3, 956072. | No data on prevalence/risk of AP in IBDs patients |
| 4. Johannsen, A., Fored, M. C., Håkansson, J., Ekbom, A., & Gustafsson, A. (2015). Consumption of dental treatment in patients with inflammatory bowel disease, a register study. *PloS one*, *10*(8), e0134001. | No data on prevalence/risk of AP in IBDs patients |

**Table S4.** Quality of evidence by GRADE approach

| **Certainty assessment** | | | | | | | **No. patients** | | **Relative Effect** | **Certainty of evidence (GRADE)** |
| --- | --- | --- | --- | --- | --- | --- | --- | --- | --- | --- |
|  | | | | | | | | | | |
| **No of studies** | **Study design** | **Risk of bias** | **Inconsistency** | **Indirectness** | **Imprecision** | **Other considerations** | **IBDs** | **Control** | **OR (95%CI)** |  |
| **Outcome. Association IBDs-AP at patient level** | | | | | | | | | | |
| 5 | Observational | Serious | Not serious | Not serious | Not serious | None | 124/208  (59.6%) | 157/281  (55.87%) | 1.57  (1.04 to 2.35) | ⨁⨁◯◯  Low |
| **Outcome. Association IBDs-AP at tooth level** | | | | | | | | | | |
| 2 | Observational | Serious | Serious | Not serious | Serious | None | 172/2158  (7.97%) | 316/4984  (6.34%) | 1.91  (1.16 to 3.15) | ⨁◯◯◯  Very low |

*Abbreviations. CI: Confidence Interval; No: number; OR: Odds Ratio.*


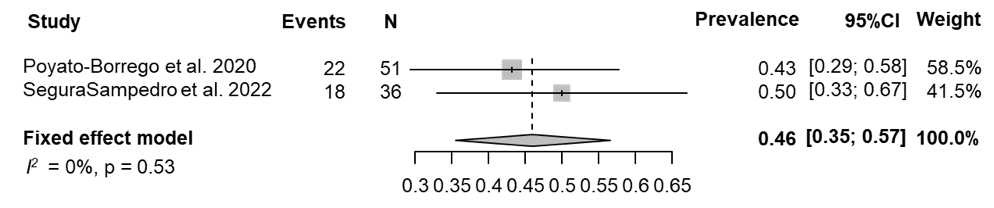
**Figure S1.** Overall prevalence of RFT-AP - Patient level.

**Figure S2.** Prevalence of RFT-AP in the IBDs and control group/healthy subjects - Patient level.


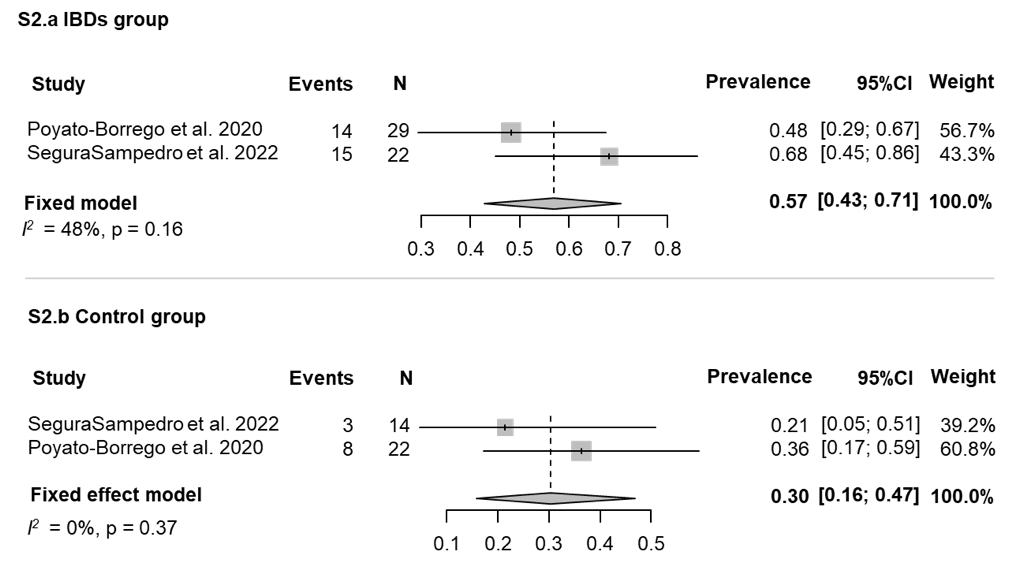


**Figure S3.** Pooled OR for RFT-AP in IBDs/healthy subjects - Patient level.


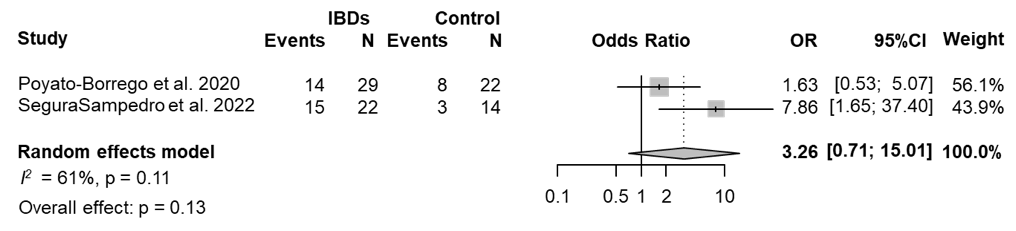


**Figure S4.** Overall prevalence of AP - Tooth level.


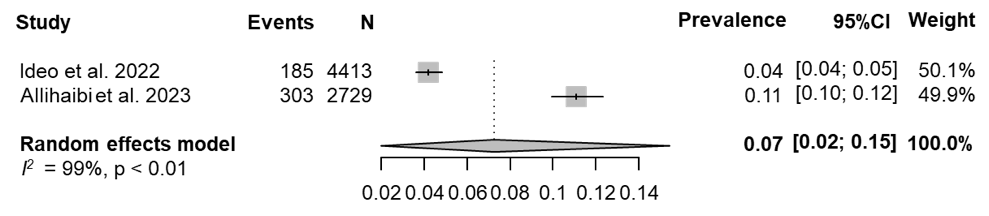


**Figure S5.** Prevalence of AP in the IBDs and control group/healthy subjects – Tooth level.


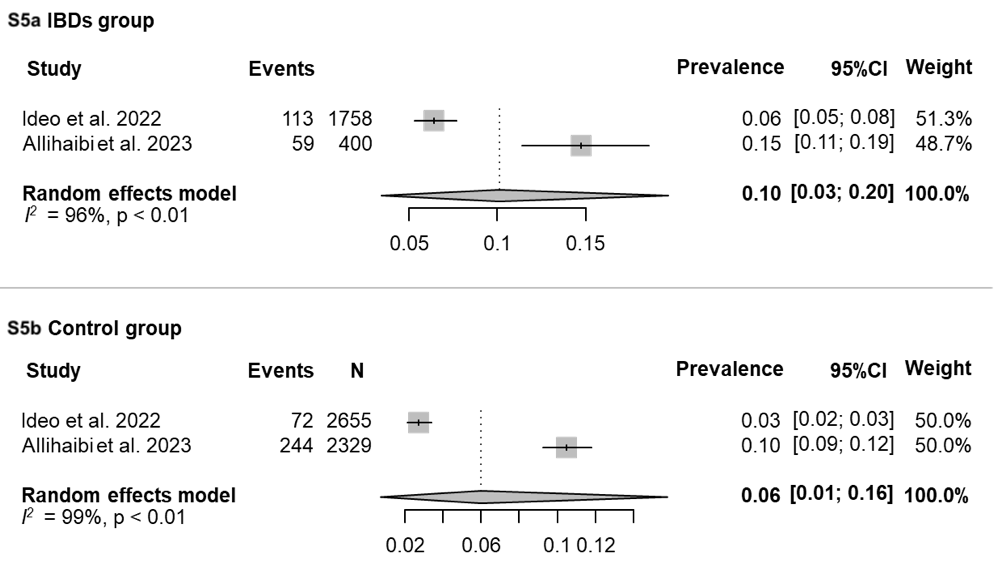


**Figure S6.** Pooled OR for AP in IBDs/healthy subjects - Tooth level.


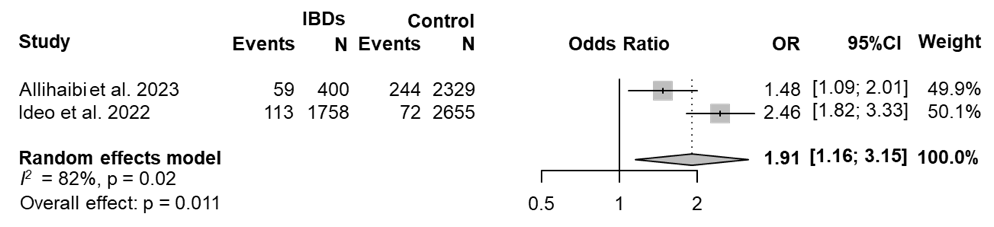


**Figure S7.** Joanna Briggs Institute critical (JBI) appraisal checklist for cohort studies (retrospective) and case-control studies.


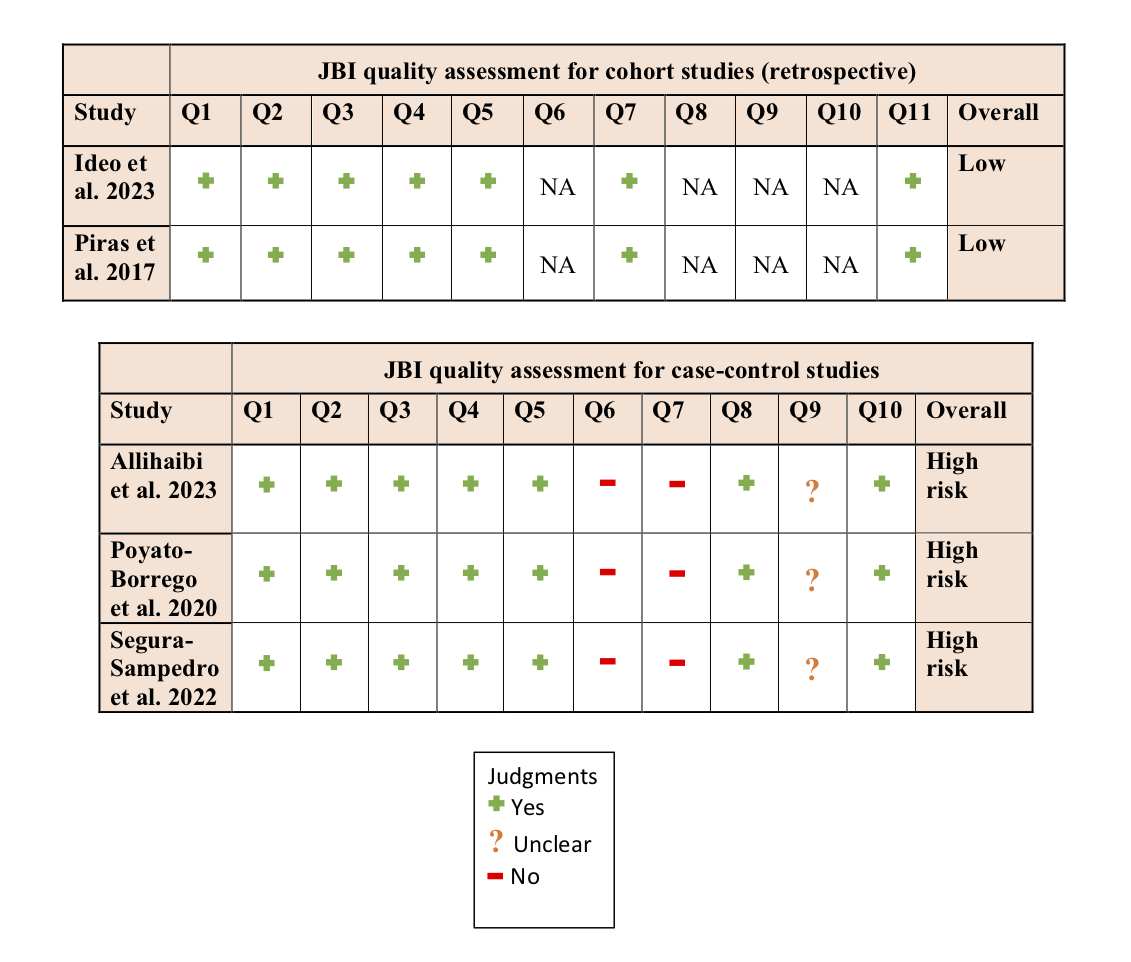

Supplement: Supplementary file 1 [file Supplementaryfile1.docx]
